# Targeting the Epigenome in Colorectal Cancer

**DOI:** 10.3390/ijms27146486

**Published:** 2026-07-21

**Authors:** Antonios N. Gargalionis, Kostas A. Papavassiliou, Athanasios G. Papavassiliou

**Affiliations:** 1 Department of Biological Chemistry, Medical School, National and Kapodistrian University of Athens, 11527 Athens, Greece; 2 First University Department of Respiratory Medicine, ‘Sotiria’ Chest Hospital, Medical School, National and Kapodistrian University of Athens, 11527 Athens, Greece; konpapav@med.uoa.gr

**Keywords:** colorectal cancer, epigenetic therapy, drug resistance, immunotherapy sensitization, epigenetic reprogramming, DNA methylation, histone modifications

## Abstract

Epigenetic alterations promote colorectal cancer (CRC) development, plasticity, and drug resistance. Agents have been developed to target epigenetic modifiers; however, they demonstrate limited clinical efficacy. This outcome is the result of the complex interplay within the epigenome, as well as that of the molecular circuits linking these epigenetic vulnerabilities with genetic mutations, oncogenic pathways, and activation of transcription factors. Therefore, current evidence suggests these agents should be assessed in combination with regimens including additional epigenetic drugs, immune checkpoint inhibitors, monoclonal antibodies, and chemotherapeutic drugs. Herein, we highlight recent advances towards epigenome-centered treatment strategies in CRC. We also prioritize potential efforts of epigenetic-associated therapeutic modalities, which should be further developed following integration of respective biomarkers and as tools of therapeutic reprogramming in context-dependent cellular states.

## 1. Introduction

Colorectal cancer (CRC) remains one of the most frequent malignancies worldwide, ranking as the third most diagnosed cancer and the second leading cause of cancer-related deaths [1,2]. Despite recent advancements in surgical and drug-based therapies, including targeted anti-epidermal growth factor receptor (EGFR), anti-vascular endothelial growth factor (VEGF) therapies, and immunotherapy, metastatic disease still presents a dismal prognosis. The main causes are intratumoral heterogeneity and acquired drug resistance, both limiting durable clinical responses [3,4]. For decades, research has focused on the role of genetic mutations, including *Adenomatous Polyposis Coli* (*APC*), *Kirsten Rat Sarcoma* (*KRAS*), *Tumor Protein p53* (*TP53*), and *B-Rapidly Accelerated Fibrosarcoma* (*BRAF*) in CRC development, progression, and response to treatment. However, genetic alterations do not fully explain the capacity of cancer cells to shift between proliferative, invasive, stem-like, immune-evasive, and drug resistance states.

In this context, it has been further documented that epigenetic alterations are implicated in the formation, progression, and metastasis of CRC [5,6]. Epigenetic alterations are heritable but reversible changes that regulate gene expression. Unlike genetic mutations, epigenetic modifications do not alter the underlying DNA sequence. The principal epigenetic mechanisms include DNA methylation, histone modifications, chromatin remodeling, and non-coding RNAs. DNA methylation is catalyzed by DNA methyltransferases (DNMTs) and generally suppresses gene expression when occurring at promoter CpG islands. Among histone modifications, histone acetylation and methylation are the best characterized mechanisms regulating chromatin accessibility and transcriptional activity. These alterations involve the addition or removal of acetyl or methyl groups on specific histone residues, thereby regulating transition between transcriptionally suppressive heterochromatin and active euchromatin status. Key enzymes regulating these activities are histone acetyltransferases (HATs), histone deacetylases (HDACs), histone methyltransferases (HMTs) and histone demethylases (HDMs) [7].

The entire array of these alterations, collectively termed the epigenome, represents a critical regulator of CRC cells’ identity and newly developed strategies against its key players are being investigated for future therapeutic applications [8]. In the present article, we discuss the clinical relevance of epigenetic therapy in CRC, which no longer simply represents approaches of reactivating tumor suppressor genes. The epigenome-centered targeting strategies should be evaluated as tools for therapeutic reprogramming of the anti-tumor immune response, phenotypic plasticity, drug resistance, and sensitization to chemotherapy [3,4].

## 2. Epigenetic Crosstalk in CRC

Epigenetic alterations arise early in CRC and often outnumber genetic alterations [6]. Aberrant DNA methylation is responsible for silencing of tumor suppressor genes and activation of oncogenes. Recent studies unveiled specific epigenome-associated tumor characteristics. Genome-wide DNA methylation status has been associated with inherited CRC, Lynch syndrome, and familial adenomatous polyposis (FAP), as well as with the level of responsiveness to certain treatments in sporadic CRC, mainly regarding the role of high methylation as a negative predictive biomarker of anti-EGFR treatment in metastatic CRC [9,10]. Li et al. have recently suggested that DNA methylation profile is distinct for early-onset CRC in certain racial/ethnic minorities [11]. Evident DNA methylation signatures have also been revealed across the adenoma–carcinoma sequence that characterizes CRC, thereby representing potential biomarkers to distinguish different stages of CRC oncogenesis [12]. The emergence of such epigenetic profiles proposes DNA methylation as a universal feature of CRC biology, with separate patterns that influence phenotypes and stages of the disease.

Notably, widespread DNA hypermethylation of a specific subset of cytosine (C)-guanine (G) dinucleotides (CpG) regions, known as CpG islands, is termed the CpG island methylator phenotype (CIMP) and it is crucial in CRC subsets. Studies have identified a key classification panel that characterizes CIMP-positive tumors and consists of *CACNA1G*, *IGF2*, *NEUROG1*, *RUNX3*, and *SOCS1* methylated genes. CIMP-positive classification requires methylation of ≥3/5 of these markers. These tumors predominantly incorporate *BRAF^V600E^* mutations, vast promoter methylation, and suppression of the DNA mismatch repair protein gene *MutL homolog 1* (*MLH1*) [13]. Further studies have expanded this concept by stratifying tumors into CIMP-high, CIMP-low, and CIMP-0 groups. CIMP-high tumors incorporate methylation in ≥6/8 loci; CIMP-low tumors are associated with *KRAS* alterations and present methylation in 1–5/8 loci, whereas the CIMP-0 group demonstrates no promoter methylation of the aforementioned marker molecular signature [13,14]. *BRAF^V600E^* is not only co-detected with CIMP but is also responsible for maintaining epigenetic changes that favor tumor aggressiveness, including the preservation of the CIMP itself and histone modifications of the polycomb repressive complex 2 (PRC2), a known epigenetic transcriptional repressor [15]. This interdependency substantiates that epigenetic marks are not engaged in independent events, but rather they interconnect with other types of epigenetic and genetic alterations.

Histone acetylation, methylation, and phosphorylation represent epigenetic chemical modifications which have been extensively investigated in CRC. Demonstrating a critical role in gene expression, specific histone deacetylases (HDACs), histone acetylation, and methylation marks have been recently highlighted as modulators of differentiation and plasticity in colorectal tumorigenesis [5]. Enhancers under H3K4 trimethylation are abundant in CRC tissues. They are regulated by the mixed-lineage leukemia 1 (MLL1) HMT and are rich in activator protein (AP-1)/c-Jun transcription factor (TF) binding motifs [16]. These modifications do not act alone but they show a dynamic crosstalk with DNA methylation. Mechanistically, suppressing DNA methylation in highly methylated *BRAF^V600E^* tumors fails to achieve transcriptional reactivation due to the adaptive upregulation of H3K27 and H3K4 trimethylation marks [17]. Such observations indicate that targeting epigenetic modifications with single agents poses a risk of concomitant development of adaptive oncogenic epigenetic alterations and this should be considered when designing treatment strategies.

The strong epigenetic impact on CRC development and progression is also attributed to the SWItch/Sucrose Non-Fermentable (SWI/SNF) multi-subunit chromatin-remodeling enzymes. *SWI/SNF* genes exhibit mutations in CRC, among other cancers, affecting certain subunits with tumor-suppressive role [18]. One of them, *AT-rich interaction domain 1A* (*ARID1A*), is frequently mutated in human cancers and its intact function is vital for suppressing tumor growth in CRC by triggering T-cell immune response [19].

Beyond the impact that these distinct epigenetic mechanisms have on chromatin accessibility and transcriptional potential, chromatin remodeling, histone modifications, and DNA methylation converge on driving tumor cell plasticity. Plasticity is the ability of tumor cells to transition between multiple phenotypic states. This heterogeneity is responsible for cancer cell stemness, immune evasion, and drug resistance [20]. Therefore, epigenetic involvement also includes the maintenance of CRC plasticity, a phenotypic switch that promotes CRC aggressiveness and contributes to tumor heterogeneity and progression. Integrated stress response, an adaptive process for cell survival against adverse conditions, induces CRC cell plasticity and subclonal heterogeneity by shaping sustained interferon signaling in conjunction with epigenomic and transcriptional reprogramming [3]. Epigenetic plasticity initiates transcriptional programs in response to external cues, and the subsequent heterogeneity in CRC is regulated by AP-1 and nuclear factor-kappa beta (NF-κB), both known as CRC-promoting TFs, which induce the establishment of inflammatory and regenerative cell states [4,21]. The interplay of these epigenetic mechanisms marks several biological features in CRC tumorigenesis, such as invasion, heterogeneity, cell plasticity, cell stemness, and drug resistance, posing the epigenome as an appealing aspect of CRC therapeutic strategies.

## 3. Limited Clinical Efficacy of Single-Agent Epigenetic Therapy

Epigenome-centered treatment approaches in CRC target mainly DNA methylation, histone modifications, and chromatin status. Several classes of these drugs have been evaluated in preclinical and clinical studies, including DNMT inhibitors (e.g., azacitidine, decitabine, and zebularine), HDAC inhibitors (e.g., vorinostat, panobinostat, belinostat, and resminostat), and histone methyltransferase inhibitors (e.g., EZH2, DOT1L) [22]. Nevertheless, despite encouraging preclinical findings, these approaches have shown limited clinical activity in CRC [23,24,25,26]. These observations highlight the need for epigenetic drugs to be further evaluated in context-specific tumor cell states and as tools that could improve therapeutic responsiveness.

DNMT inhibitors are approved for certain hematologic malignancies. Their primary action is to block catalytic activity of DNMTs and reactivate the expression of tumor suppressor genes. Mechanistically, DNMT inhibitors trigger the viral defense pathway by releasing the methylation-associated repression on endogenous retroviruses, thereby upregulating immune signaling and sensitizing subjects to treatment with immune checkpoint inhibitors (ICIs) [27]. Strategies targeting histone chemical modifiers encompass natural and synthetic inhibitors with a subgroup already reaching clinical trials. These agents act on HDACs, HATs, HMTs, and protein kinases; however, there are still limitations to overcome, mainly adverse effects, off-target effects, lack of selectivity, toxicity, variable clinical efficacy, and drug resistance [28].

Specifically, although *BRAF^V600^* mutation-bearing tumors in CRC have a strong association with DNA hypermethylation, it seems that DNMT inhibition does not have the anticipated clinical efficacy. DNMT inhibition with 5-azacitidine reduces DNA methylation but does not restore gene transcription. This is the result of an adaptive mechanism that accompanies drug-induced DNA hypomethylation by reducing histone acetylation and enhancing histone methylation through potentiation of the polycomb repressor complex (PRC) around the DNA hypomethylated genome sites [17]. An additional hypothesis regarding the inefficacy of single epigenetic drugs is that epigenetic alterations in CRC do not constitute oncogenic drivers of the disease. Accordingly, targeting aberrant epigenetic function alleviates transcriptional silencing by restoring levels of histone acetylation and methylation, as well as those of DNA methylation; however, this does not have a profound effect on tumor regression [17,23,24,25,26]. This poor therapeutic outcome can also be attributed to the fact that CRC is characterized by tumor heterogeneity and cancer cell plasticity; therefore, the profile of epigenetic alterations does not constitute an intratumoral universal feature, but rather the different transcriptional programs that shape certain states of cell populations [3,4].

## 4. Future: Combination Epigenetic Therapy

The limited clinical efficacy of single epigenetic agents and evidence from recent preclinical studies have shifted therapeutic development toward combination strategies (Table 1). Firstly, combination of epigenetic drugs that target different epigenetic marks seems to have a synergistic effect on tumor cell properties. Since DNMT inhibition is followed by the accumulation of H3K27me3, a histone mark regulated by the HMT enhancer of zeste homolog 2 (EZH2), combined action of DNMT inhibition with EZH2 inhibition induces activation of previously downregulated tumor suppressor genes. This synergy attenuates CRC cell proliferation and mediates active transcription through the calcium-calcineurin-nuclear factor of activated T-cells (NFAT)/AP-1 signaling pathway [29]. Additionally, combination of DNMT inhibition with HDAC inhibition significantly suppresses cell proliferation and cell cycle progression, and triggers apoptosis in colorectal adenocarcinoma. This is also accompanied by the re-expression of a number of tumor suppressor genes [30]. Thus, the concomitant administration of epigenetic agents according to the tumor’s epigenetic molecular profile could ameliorate clinical efficacy (Table 1).

Epigenetically regulated transcription factors (TFs)/cofactors (coactivators/corepressors) play an important role in CRC development and progression. This makes them valuable therapeutic targets. As TFs are positioned at the convergence of tumorigenic signal transduction pathways and are decisive for abnormal gene expression, blocking their activity will probably result in the successful inhibition of tumor cell hallmarks without CRC cells being able to easily develop bypass mechanisms of drug resistance. Consequently, drugs targeting epigenetic features of TFs and/or their cofactors may also be combined with other agents for more efficient management of CRC in the clinical setting [31].

Aberrant epigenetic alterations, including dysregulation of histone acetylation and/or DNA methylation, may contribute to the establishment of an immunosuppressive tumor microenvironment (TME) through context-dependent regulation of immune-related gene expression. Hampering these modifications sensitizes tumors to ICIs [32]. HDAC inhibitors, in particular, mediate immune anti-cancer effects, thereby modulating the efficacy of immunotherapeutic drugs. HDAC inhibitors produce a synergistic effect with ICIs to regulate the TME [33]. Most of the advanced CRC tumors have a microsatellite stable/proficient mismatch repair (MSS/pMMR) profile and in these tumors the combined inhibition of HDACs and indoleamine 2,3-dioxygenase 1 (IDO1; a heme-containing enzyme that catalyzes the initial rate-limiting step in the degradation of the essential amino acid tryptophan along the kynurenine pathway) influences the immune microenvironment by triggering tumor immunogenic cell death, enhancing CD8+ T cell infiltration in vitro and in vivo [34]. In the ECHO-206 phase I/II study, patients with advanced solid cancers were administered azacitidine along with pembrolizumab and IDO1 inhibitor. The regimen was well-tolerated but did not reach substantial clinical response [35]. The CAPability-01 trial combined the anti-programmed cell death protein-1 (PD-1) antibody sintilimab with chidamide, which is a HDAC inhibitor. This combination was administered in patients with advanced or metastatic CRC, frequently harboring MSS/pMMR profile with or without bevacizumab. The triplet treatment, including sintilimab, chidamide, and bevacizumab, demonstrated improved progression-free survival (PFS), overall response, and median PFS rate. These patients exhibited higher CD8+ T cell infiltration in RNA sequencing and, thereby, improved immune-based TME [36]. When pembrolizumab was combined with the DNMT inhibitor CC-486 (oral azacitidine) and the HDAC inhibitor romidepsin in a similar MSS/pMMR subset of CRC patients, there was a minimal clinical effect of a durable partial response and stable disease in two patients, highlighting the complexity of combining immunotherapy with epigenetic agents [32].

Beyond immunotherapy, epigenetic agents are also being assessed in combination with conventional chemotherapy. The study by Calibasi-Kocal et al. evaluated the therapeutic potential of quisinostat, a multi-HDAC inhibitor, with 5-fluorouracil (5-FU) in CRC cell lines. HDAC inhibition significantly restores H3K27 acetylation but also increases cytotoxicity induced by 5-FU. Furthermore, the combination acts in synergy to suppress cell proliferation, induce apoptosis, impede cell migration, and attenuate epithelial-to-mesenchymal transition (EMT) in vitro. This implies that HDAC inhibitors may have additive effects on chemotherapy in CRC [37].

Hypomethylating agents have also been evaluated in combination with anti-EGFR monoclonal antibodies (mAbs). Combination of the hypomethylating agent decitabine and the anti-EGFR mAb panitumumab has been well-tolerated and demonstrates effect on wild-type *KRAS* patients with metastatic CRC, who were previously treated with cetuximab [23]. Beyond these clinical combination studies, recent preclinical data have identified additional epigenetic strategies that modulate tumor cell plasticity and differentiation programs. Epigenetic mechanisms are responsible for the dynamic alteration of differentiation states that characterize cellular plasticity and disease heterogeneity, which foster acquired resistance. In this context, the administration of EZH2 inhibitor sensitizes *KRAS*-mutant CRC cells to inhibitors of the Ras pathway. Combined inhibition of EZH2 and Ras pathway components promotes anti-tumor effects and decreases tumor growth. This is accompanied by enhanced differentiation via upregulation of the Wnt pathway repressor transducin-like enhancer protein 4 (TLE4) [38]. Another promising preclinical strategy targets the interaction between EZH2 and the farnesoid X receptor (FXR). FXR, a ligand-activated TF and nuclear receptor, is an additional target of EZH2. FXR deficiency promotes CRC and a developed FXR agonist displays consequent tumor inhibition. EZH2 transcriptionally silences FXR in CRC through H3K27me3, therefore the combination of FXR pharmaceutical upregulation with EZH2 inhibition demonstrates anti-tumor effects by ameliorating FXR nuclear translocation in vitro (Table 1) [39].

## 5. Concluding Remarks—Outlook

Collectively, the evidence discussed above highlights the epigenome as an intricate network encompassing DNA methylation, histone modifications, and chromatin rearrangements that regulate CRC progression, aggressiveness, and therapeutic resistance. Although representative epigenetic agents have shown promising potential in preclinical studies, their translation into clinically effective monotherapies for CRC remains limited. Epigenetic targeting in CRC seems also inefficient when included in strategies strictly aiming to restore epigenetic silencing. These observations form current approaches to address epigenetic mechanisms as epigenetic vulnerabilities in context-dependent cellular states rather than aberrant epigenetic alterations. Moreover, epigenetic mechanisms regulate cellular plasticity, a dynamic and reversible process; hence, epidrugs can be evaluated as tools of therapeutic reprogramming. Inhibitors of DNA methylation, histone deacetylation, and histone methylation should be tested in biomarker-driven settings as components of combination treatments with chemotherapy, mAbs, and immune checkpoint blockade. Future advances are likely to integrate epigenomic biomarkers and precision therapeutics, to shift the approaches from horizontal epigenetic modulation toward context-dependent therapeutic manipulation.

## Figures and Tables

**Table 1:** Strategies for the Rational Use of Epigenetic Agents in Colorectal Cancer.

Strategy	Core Concept	Key Therapeutic Implication	References
DNMT + EZH2 inhibition	EZH2 inhibition restores H3K27me3-dependent transcription after DNMT inhibition	Reactivation of tumor suppressor genes and enhancement of transcriptional reprogramming	[29]
DNMT + HDAC inhibition	Combined reversal of DNA methylation and histone deacetylation	Synergistic inhibition of proliferation and induction of apoptosis	[30]
Epigenetic targeting of TF activity	Targeting epigenetic regulation of TFs in combination with chemotherapy or targeted therapy	Selective targeting of TFs to overcome adaptive transcriptional resistance	[31]
Epigenetic therapy + ICIs	Epigenetic targeting enhances tumor immune signaling and remodels the tumor immune microenvironment	Sensitization of specific tumor subtypes to immunotherapy	[27,32,33,34,35,36]
HDAC inhibition + chemotherapy	HDAC inhibition has additive anti-tumor effects on 5-FU chemotherapy in vitro	Increased apoptosis and reduced EMT	[37]
DNMT inhibition + anti-EGFR	Reversal of epigenetic silencing by DNMT inhibition and sensitization to anti-EGFR blockade	Well-tolerated with anti-tumor effects, partial responses in previously cetuximab-treated patients, disease stabilization in additional patients	[23]
EZH2 + Ras inhibition	Enhancement of differentiation via TLE4 upregulation	Sensitization of *KRAS*-mutant CRC cells to Ras pathway inhibition	[38]
EZH2 + FXR agonist	Reversal of H3K27me3-mediated FXR silencing by EZH2 inhibition, promotion of FXR nuclear localization, and CDX2 upregulation by FXR agonist	Synergistic inhibition of CRC clonogenic growth, invasion in vitro and attenuated tumor growth in vivo	[39]

Abbreviations: CRC, colorectal cancer; DNMTs, DNA methyltransferases; EGFR, epidermal growth factor receptor; EMT, epithelial-to-mesenchymal transition; EZH2, enhancer of zeste homolog 2; FXR; farnesoid X receptor; HDACs, histone deacetylases; H3K27me3, H3K27 trimethylation; ICIs, immune checkpoint inhibitors; TF, transcription factor; TLE4, transducin-like enhancer protein 4; 5-FU, 5 fluorouracil.

## Data Availability

No new data were created or analyzed in this study. Data sharing is not applicable to this article.
